# Two Switchable Plasmonically Induced Transparency Effects in a System with Distinct Graphene Resonators

**DOI:** 10.1186/s11671-020-03374-1

**Published:** 2020-07-03

**Authors:** Jingrui Guan, Shengxuan Xia, Zeyan Zhang, Jing Wu, Haiyu Meng, Jing Yue, Xiang Zhai, Lingling Wang, Shuangchun Wen

**Affiliations:** Key Laboratory for Micro/Nano Optoelectronic Devices of Ministry of Education & Hunan Provincial Key Laboratory of Low-Dimensional Structural Physics and Devices, School of Physics and Electronics, Hunan University, Changsha, 410082 China

**Keywords:** Plasmonically induced transparency, Graphene plasmons, Plasmonic sensing, Perfect absorption

## Abstract

General plasmonic systems to realize plasmonically induced transparency (PIT) effect only exist one single PIT mainly because they only allow one single coupling pathway. In this study, we propose a distinct graphene resonator-based system, which is composed of graphene nanoribbons (GNRs) coupled with dielectric grating-loaded graphene layer resonators, to achieve two switchable PIT effects. By designing crossed directions of the resonators, the proposed system exists two different PIT effects characterized by different resonant positions and linewidths. These two PIT effects result from two separate and polarization-selective coupling pathways, allowing us to switch the PIT from one to the other by simply changing the polarization direction. Parametric studies are carried to demonstrate the coupling effects whereas the two-particle model is applied to explain the physical mechanism, finding excellent agreements between the numerical and theoretical results. Our proposal can be used to design switchable PIT-based plasmonic devices, such as tunable dual-band sensors and perfect absorbers.

## Introduction

Surface plasmons are the collective resonating modes of free electrons that are generated at the interface between the insulating and conducting media [1, 2]. Due to their ability to confine an incident electromagnetic field to the ultimate limit size of one-atom scale at the subwavelength range [3], surface plasmons have become one of the most fundamental and important methods to achieve strong light-matter interactions [4]. This appealing optical phenomenon has been found in various types of plasmonic systems, which facilitates the development of a variety of state-of-the-art applications such as biosensing [5], nonlinear optics [6, 7], absorbers [8–11], and other plasmonic modulators [12–15]. The possibilities to achieve these significant applications are attributed to some interesting phenomena like plasmonically induced transparency (PIT). The process known as PIT is a consequence of the near-field coupled Fano interference and is featured by the generation of a prominent window in an optical spectrum as it eliminates the resonant absorption in the system. During the past years, such coherent plasmonic interaction has been used to achieve a variety of applications such as plasmonic switching [16], slow light propagation [17] and sensing [18], and optical storage [19].

Though recent studies have revealed that ultrathin metal films down to atomic thickness can possess dynamic electrical tunability [20, 21], plasmons supported by these novel metals still suffer from relatively large ohmic and radiative losses of the metals [22, 23]. Such shortcoming of the metals limits the further development of metal-supported PIT, and it is necessary to find new plasmonic materials. In contrast to metallic plasmons, plasmons supported by graphene (a single atomic layer of tightly structured carbon atoms formed to a symmetric hexagonal honeycomb lattice) can not only be continuously and dynamically tuned through electrostatic biasing [24, 25], but also have long propagation length, which enables a new generation of restructurable plasmonic devices and, thus, provides an ideal platform to achieve active PIT [26, 27]. Although various materials and designs have been used to achieve PIT in pure metal [16, 28–31] and graphene [32–42], or their hybrid material-based [43–45] systems, most of these systems can only realize single PIT effect. For example, one of the common ways to achieve PIT is to design *π*-shaped/like metasurfaces [16, 28, 30, 33, 37, 45]. Another way is to construct grating-coupled systems [32, 34]. However, these kinds of structures can only realize polarization-dependent single-window PIT. This is because, due to the special geometrical asymmetry of these structures, all of the nanostructured resonators are preset to operate as either the bright (radiative/superradiant) or the dark (nonradiative/subradiant) mode. Therefore, they only allow one bright to dark coupling pathway in one particular polarization direction, resulting in only one polarization-dependent PIT effect. Though our previous studies have demonstrated PIT systems with two bright-dark mode coupling pathways in pure graphene nanoribbons (GNRs) [35] or grating-coupled [38] structures, the tally polarization-insensitive single-window PIT or polarization-dependent double-window PIT effects in these systems strongly dependent on the particular choice of the geometrical parameters (see discussion part).

In this paper, we propose to use two distinct resonators, namely GNRs couple with a graphene sheet loaded with dielectric gratings, to couple and realize two separate PIT effects. We will demonstrate that by setting perpendicular resonating directions, surface plasmons that resonate in both resonators will be generated under different polarization directions of the incident light, resulting in two different polarization-dependent coupling pathways and, therefore, two separate PIT effects. Besides, parametric studies will be used to investigate in detail the coupling mechanisms. And both advanced simulations and two-particle model-based theoretical analyses will be combined to demonstrate these switchable PIT effects. Finally, the potential applications of the proposed system, such as refractive index sensors and perfect absorbers, and the comparations with other PIT systems will be discussed.

## Designs and Materials

**Fig. 1:**
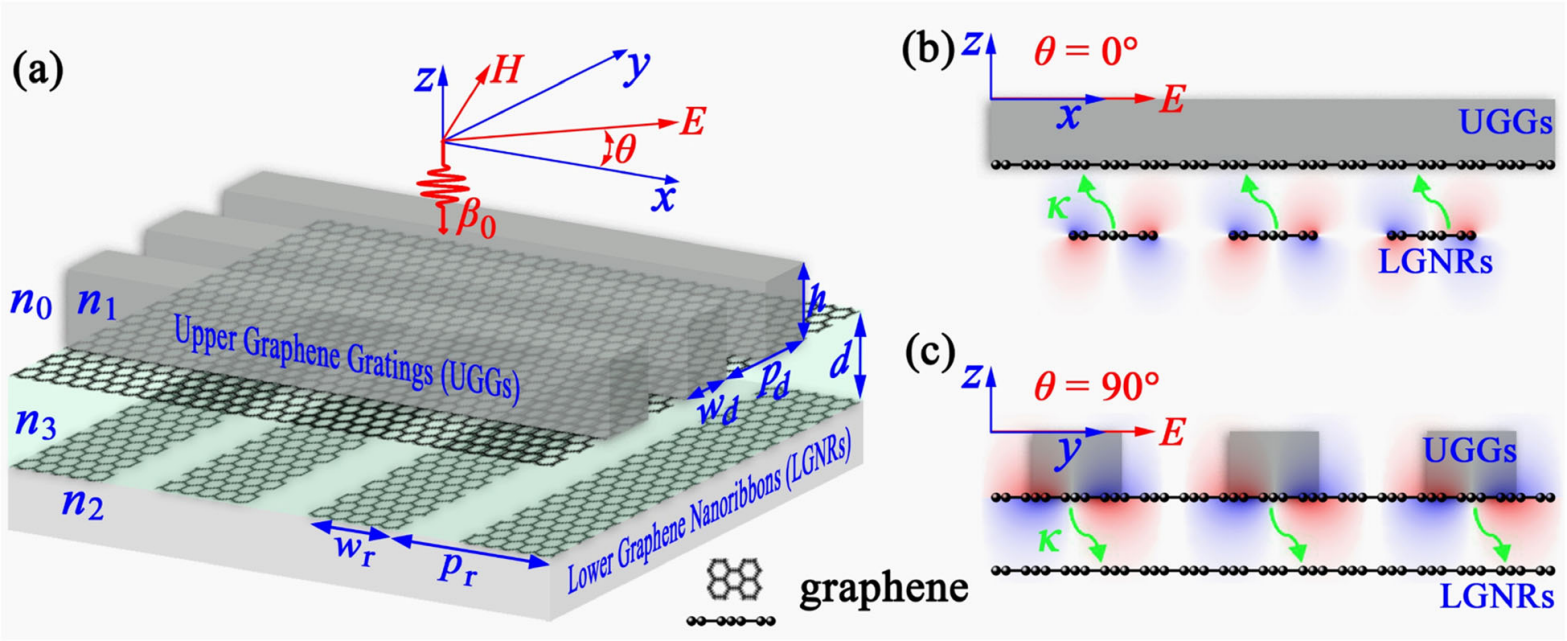
3D (**a**) and 2D side views (**b**, **c**) of the proposed PIT system. One graphene layer loaded with periodic dielectric grating covers above one layer of periodic GNRs with crossed grating and ribbon directions. The upper graphene grating layer is designed with grating width *W*_*d*_, height *h*, and transverse period *P*_*d*_, while the lower graphene nanoribbons have ribbon width *W*_*r*_ and period *P*_*r*_. The distance between the two graphene layers is *d*, which is assumed to be filled by a conductive Si or SiO_2_ spacer with refractive index *n*_3_. The refractive indices of and over the dielectric gratings and bellow the GNRs are labeled as *n*_1_, *n*_0_, and *n*_2_, respectively. A linearly polarized plane wave with wave number *β*_0_ and polarization angle *θ* with respect to the *x*-axis normally impinges the surface of the layered graphene system

In this part, we introduce the numerical model and the related materials used in this study. We state that in our model, we only consider the classical electrodynamics and neglect any effects that may arise from the possible quantum finite-size effects of GNRs, nonlinear effects of graphene, and substrate phonons effects [46]. The schematic of the proposed system is shown in Fig. 1. Two graphene layers are placed on the *x*o*y* plane and separated by a doped Si or SiO_2_ conductor with refractive index *n*_3_ and thickness *d*. The first layer is formed by lower graphene nanoribbons (LGNRs) with a period in the *x* direction. The second layer is a whole graphene sheet, which is further covered by dielectric gratings with a refractive index *n*_1_ (labeled as upper graphene gratings, UGGs) and a period *P*_*d*_ in the *y* direction. The geometric parameters are fixed as *W*_*r*_ = *W*_*d*_ = 50 nm, *p*_*r*_ = *p*_*d*_ = 100 nm, *h* = 100 nm, and *d* = 20 nm, as defined in Fig. 1. The dielectric constants of the other materials surrounding the graphene layers are set to *n*_0_ and *n*_2_, shown in Fig. 1. For simplicity and without loss of generality, the dielectric constants are assumed to be *n*_1_ = 2.0 and *n*_0_ = *n*_2_ = *n*_3_ = 1.0. The neglecting of the imaginary part of the refractive index would not change the fundamental conclusions of this study. Note that the above parameters keep the same unless otherwise specified. Technologically, the realization of the designed two-layer graphene-based PIT system is experimentally feasible using the well-developed patterning and grating techniques, which has been recently used to fabricate the layered graphene system [27, 47].

## Methods

The proposed structure is numerically simulated by using the finite-difference time-domain (FDTD) method based on Lumerical FDTD Solutions. In our simulations, periodic boundary conditions are used in both x and y directions. Broadband plane waves are incident from the z direction, along which perfectly matched layers are applied to absorb all the light coming out to the boundaries. Electrical field distribution is gathered by 2D field profile monitors at the resonant wavelength with a 0.5 nm distance to the graphene surface between the two layers. Besides, the graphene film is described within the random-phase approximation (RPA) [48, 49]. Within this approximation, the in-plane optical conductivity *σ* of graphene is written as a semiclassical Drude-like expression in the mid-infrared range as *σ*(*ω*) = *ie*^2^*E*_*F*_/[*πћ*^2^(*ω* + *iτ*^−1^)] [24, 50]. Here, *E*_*F*_ = *ћν*_*F*_(*n*_*g*_*π*)^1/2^ is the Fermi level of graphene, with *n*_*g*_*=* (*μ/ћν*_*F*_)^2^*/π* being the carrier concentration (where *μ* = 15,000 cm^2^/(*V* × *s*) is the measured dc mobility, *ν*_*F*_ = 10^6^ m/s is the Fermi velocity, and *ћ* is the reduced Plank’s constant) and can be tuned by electrical gating [24, 25, 50], *ω* is the angular frequency, and *τ = μE*_*F*_*/*(*ev*_*F*_^2^) is the carrier relaxation time. In this paper, *E*_*F*_ is fixed to 0.6 eV unless otherwise specified. In our simulations, the optical property of graphene is described by using an anisotropic relative permittivity tensor [35]. The *z* component of graphene permittivity is set as *ε*_*zz*_ = 2.5 based on the dielectric constant of graphite, whereas the in-plane components are *ε*_*xx*_ = *ε*_*yy*_ = 2.5 + *iσ*(*ω*)/(*ε*_0_*ωt*) [24, 51], with *ε*_0_ is the vacuum permittivity and *t* = 1 nm is the thickness of graphene [35].

By applying the two-particle model in both of the *x* and *y* axes, we can theoretically analyze the effective plasmonic resonances and couplings shown in Fig. 1 by the following set of equations [8, 52, 53]:

1 $$ {a}_{1i}^{{\prime\prime} }(t)+{\gamma}_{1i}{a}_{1i}^{\prime }(t)+{\omega}_{1i}^2{a}_{1i}(t)+{\kappa}_{12i}^2{a}_{2i}(t)={Q}_{1i}E\sin \theta /{m}_{1i} $$

2 $$ {a}_{2i}^{{\prime\prime} }(t)+{\gamma}_{2i}{a}_{2i}^{\prime }(t)+{\omega}_{2i}^2{a}_{2i}(t)+{\kappa}_{21i}^2{a}_{1i}(t)={Q}_{2i}E\cos \theta /{m}_{2i} $$

where *i* = *x* or *y*; *γ*_*i*_ is the loss factor, which relates to the linewidth of the spectrum; *ω*_*i*_ is the resonance frequency of the resonator; *Q*_*i*_ is the effective charges of the modes, which show the strength of the resonant mode; and *m*_*i*_ is the effective masses of the particles in corresponding resonant orientation. *κ*_*i*_ is the coupling strength between the two layers in the *i* direction, which corresponds to the electron-electron interactions of the two coupled modes and, therefore, is determined by the special field distribution of the plasmons and the coupling distance between the resonators. Considering that the plasmonic couplings are only along the two coordinate axes with coupling strength *κ*_12*i*_ = *κ*_21*i*_ = *κ*_*i*_, we can treat the system as two separate groups of resonators resonating independently at different directions. We assume all particles couple with the incident electric field *E* = *E*_0_*e*^*iωt*^, generating the displacement vectors *a*_*i*_ = *c*_*i*_*e*^*iωt*^. After performing some algebraic calculations on Eqs. (1) and (2), the mode amplitudes of plasmons can be expressed as:

3 $$ {a}_{1i}(t)=\frac{\kappa_i^2{Q}_{2i}E\cos \theta /{m}_{2i}+\left({\omega}^2-i{\omega \gamma}_{2i}-{\omega}_{2i}^2\right){Q}_{1i}E\sin \theta /{m}_{1i}}{\kappa_i^4-\left({\omega}^2-i{\omega \gamma}_{1i}-{\omega}_{1i}^2\right)\left({\omega}^2-i{\omega \gamma}_{2i}-{\omega}_{2i}^2\right)} $$

4 $$ {a}_{2i}(t)=\frac{\kappa_i^2{Q}_{1i}E\sin \theta /{m}_{1i}+\left({\omega}^2-i{\omega \gamma}_{1i}-{\omega}_{1i}^2\right){Q}_{2i}E\cos \theta /{m}_{2i}}{\kappa_i^4-\left({\omega}^2-i{\omega \gamma}_{1i}-{\omega}_{1i}^2\right)\left({\omega}^2-i{\omega \gamma}_{2i}-{\omega}_{2i}^2\right)} $$

The effective electric susceptibility (*χ*_eff_), which shows the ratio between the total polarizability (*P*) of the plasmonic resonators and the strength of the incident electric field, then can be expressed in forms of the displacement vectors as:

5 $$ {\displaystyle \begin{array}{c}{\chi}_{e\mathrm{ff},i}=\frac{P_i^2}{\varepsilon_0E}=\frac{Q_{1i}{a}_{1i}+{Q}_{2i}{a}_{2i}}{\varepsilon_0E}\\ {}=\frac{\left[{\kappa}_i^2{Q}_{1i}{Q}_{2i}+\left({\omega}^2-i{\omega \gamma}_{2i}-{\omega}_{2i}^2\right){Q}_{1i}^2\right]\sin \theta /{m}_{1i}+\left[{\kappa}_i^2{Q}_{1i}{Q}_{2i}+\left({\omega}^2-i{\omega \gamma}_{1i}-{\omega}_{1i}^2\right){Q}_{2i}^2\right]\cos \theta /{m}_{2i}}{\varepsilon_0\left[{\kappa}_i^4-\left({\omega}^2-i{\omega \gamma}_{1i}-{\omega}_{1i}^2\right)\left({\omega}^2-i{\omega \gamma}_{2i}-{\omega}_{2i}^2\right)\right]}\end{array}} $$

Then, the simulated transmission and absorption spectra can be fitted by the imaginary part of the susceptibility. In this paper, the absorption is defined as *A* = Im[*χ*_eff, *i*_]. This coefficient is derived from the conservation of energy relation *T* + *A* = 1; therefore, we have the expression of the transmission *T* = 1 − Im[*χ*_eff, *i*_].

## Results

### Excitation of PIT Effects

To excite the PIT effects, one problem that needs to be solved is how to design a bright mode resonator. Because of the large momentum mismatch between the incoming free-space waves and plasmon waves, the excitation of plasmons is one of the main challenges for the usage of graphene plasmons. To end this, several approaches enabling the excitation of graphene plasmons have been proposed and theoretically and experimentally demonstrated. The first commonly used method is patterning the graphene monolayer to coplanar nanostructures, such as nanoribbons [25, 54], nanodisks [55, 56], and circles [24]. Another method is to construct grating configurations in a continuous graphene sheet, which is achieved either by using the diffractive dielectric gratings [51, 57] and the electric field gates [58] to construct position-dependent periodic local conductivity gratings or by using periodic diffractive corrugation gratings formed by the graphene sheet itself [57, 59]. The reason why the surface plasmons can be excited in these graphene structures is that the nanostructures or gratings can provide the plasmon waves with an additional reciprocal wavevector which is necessary for the compensation of the wavevector mismatch when the polarization direction is along the periodic direction [51, 54, 59]. In this condition, the graphene resonator can operate as a bright mode, or it may serve as a dark mode. Here, we propose to use both of the GNRs and the graphene sheet loaded with rectangularly shaped dielectric gratings to respectively work as bright and dark modes to construct a PIT system, as shown in Fig. 1.

**Fig. 2:**
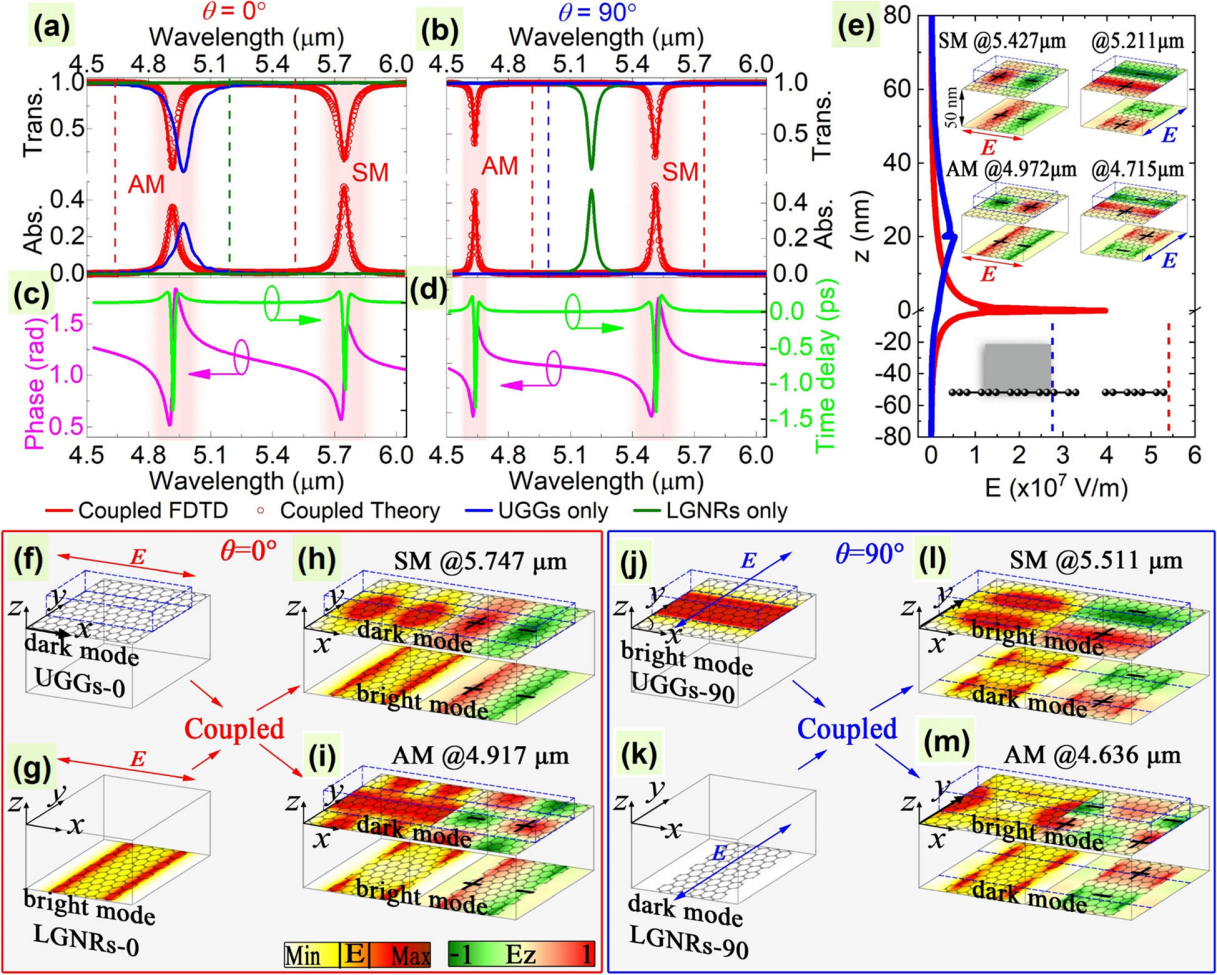
Transmission and absorption lines (**a**, **b**) and their transmission phase (left *y* axis) and delay time (right *y* axis) (**c**, **d**) of the system with polarization angle *θ* = 0° (**a**, **c**) and 90° (**b**, **d**), respectively. In (**a**) and (**b**), the dashed lines show the resonant position at the other polarization. The fitting parameters of the theoretically analyzed lines in (**a**) and (**b**) are (in THz) 6.71, 110.07, 2.25, 0.46, and 0.74, and 5.73, 4.13, 72.83, 0.33, and 0.27 for *κ*_*i*_, *Q*_*1i*_/sqrt(*ε*_0_*m*_*1i*_), *Q*_*2i*_/sqrt(*ε*_0_*m*_*2i*_), *γ*_*1i*_, and *γ*_*2i*_, respectively. Spatial distributions of the electric field for the cases with UGGs (**e** (blue line), **f**, **j**) and LGNRs (**e** (red line), **g**, k) only along the *z* axis (**e**) and in the corresponding graphene planes (**f**, **g**, **j**, **k**). Spatial distributions of the electric field (left panels) and the corresponding *z* component (right panels) of the symmetric mode (SM) (**h**, @5.747 μm; **l**, @5.511 μm) and antisymmetric mode (AM) (**i**, @4.917 μm; **m**, @4.636 μm) at polarization angles *θ* = 0° (**h**, **i**) and 90° (**l**, **m**), respectively. The upper inserts in **e** show *z* component of the electric field for the case with *d* = 50 nm, while the lower insert depicts the position of the field in the main plot. Signs “+” and “−” donate the resonating surface charges; the darker color refers to a larger charge density

To explore the mechanism of the proposed PIT system, numerical simulations of the configuration shown in Fig. 1 are calculated, and the corresponding results for two different polarization angles are shown in Fig. 2. For the case with *θ* = 0°, we first calculated the results for the situation when the lower graphene nanoribbons (LGNRs) and the upper dielectric grating-loaded graphene exist alone. Because the polarization direction is perpendicular to the LGNRs, SPs can be excited on them, resulting in a main absorption peak at 5.327 μm, as the blue line shown in Fig. 2a. In contrast, SPs on the upper graphene sheet cannot be excited on this polarization condition because the incident light is polarized parallel to the dielectric gratings, resulting in a strong momentum mismatch, as it is demonstrated by the flat green lines in Fig. 2a. In these situations, we refer the directly excited mode in the LGNRs and dark mode in the UGGs as LGNRs-0 and UGGs-0, respectively, as shown in Fig. 2g and f. However, what is interesting here is that when these two graphene layers are brought together and close enough, two absorption peaks (transmission dips) appear at 5.747 μm and 4.917 μm. The one with longer resonant wavelength is dominant by absorption reaching 47.16%, whereas the other one with shorter resonant wavelength is characterized by an absorption peak of 35.88%, indicating that these two modes interact very strongly with the external incident light, as shown in Fig. 2a and concluded in Table 1. These two modes originate from the in-phase and out-of-phase plasmonic couplings between the two resonators. Specifically, the bright mode resonance on the LGNRs should be considered as a fixed mode because it is directly excited by the incident light. However, the plasmon resonance in the upper graphene layer cannot be directly excited but can couple with those excited in the LGNRs through both the in-phase and out-of-phase interactions. It is the coexistence of the two resonators and their plasmonic couplings that directly result in this PIT effect. To reveal the physical mechanisms behind the origin of the two modes clearly, we display the electric field distributions at these two modes in Fig. 2h and i. According to the *E*_*z*_ component field distributions, the mode at 5.747 μm shows an in-phase resonance nature of the layered structure and, therefore, is called the symmetric mode. The mode at 4.917 μm shows the antiphase resonance and is called the antisymmetric mode. Besides, Fig. 2h and i clearly reveal the structure of these modes: All of the *E*_*z*_ components show a dipole mode resonance featured by a 2*π* phase shift along the polarization direction (*x* axis) in each graphene layer. These two fundamental modes eliminate the resonant absorption of the LGNRs while giving rise to a prominent transmission window and two absorption peaks in the optical spectrum, causing the optical effect called PIT (for the convenience of discussion, we call this LGNRs-PIT). In Fig. 2c, we also plot the transmission phase and the delay time at the two absorption peaks, the latter reach 0.34 ps and 0.36 ps, respectively, indicating the slowing light effect in the system.

**Table 1:** Characteristic parameters of Fig. 2a and b

Modes		Wavelength (μm)	Absorption ratio (%)	Line width (nm)	Slowing light (ps)
*θ* = 0°	Symmetric mode	5.747	47.16	51	0.34
	Antisymmetric mode	4.917	35.88	61	0.36
*θ* = 90°	Symmetric mode	5.511	49.07	34	0.23
	Antisymmetric mode	4.636	46.46	20	0.21

While for the case with *θ* = 90°, SPs can be excited on UGGs with a main absorption peak at 5.202 μm but cannot on the LGNRs when they exist alone, as the green and blue lines shown in Fig. 2b, respectively. In these situations, we refer the directly excited mode in the UGGs and dark mode in the LGNRs as LGNRs-90 and UGGs-90, respectively, as shown in Fig. 2j and k. However, when these two modes are close enough to couple with each other, two transmission dips (absorption peaks) clearly appear at 5.511 μm and 4.636 μm with absorption reaching 49.07% and 46.46%, respectively, meaning that the interactions with the external incident waves are very strong, as shown in Fig. 2b and concluded in Table 1. Similar to the case with *θ* = 0°, the physical mechanism can also be understood by considering the in-phase and out-of-phase plasmon couplings between the two graphene layers. As it is clearly illustrated by Fig. 2l and m, the *E*_*z*_ component field distributions show a dipole mode resonance featured by a 2*π* phase shift along the *y* axis in each graphene layer and reveal in-phase (symmetric mode) and out-of-phase (antisymmetric mode) resonances at the corresponding absorption peaks. It is these two fundamental modes that eliminate the resonant absorption of the case with only the UGGs while generating a prominent transmission window and two absorption peaks in the optical spectrum, resulting in another PIT (for the convenience of discussion, we call this UGGs-PIT). In Fig. 2d, the transmission phase and the delay time at the two absorption peaks are also plotted, showing the slowing light propagation effect with peak values of 0.23 ps and 0.21 ps at the symmetric mode and antisymmetric mode, respectively.

However, it is important to note that though the field distributions in the upper graphene sheet of the antisymmetric mode in Fig. 2i and m show a “multipole” resonant appearance, they are still a dipole mode as the charge oscillations also keep the nature of a dipole mode resonance along the polarization direction. The reason for the “multipole” resonant appearance is due to the strong field interference from the LGNRs. This can be understood by considering the fact that the strongest localized plasmon field in the patterned GNRs is more pronounced than that in the continuous graphene sheet [60], as shown in Fig. 2e. These “multipole” mode appearances will disappear by setting the field monitor out of the two graphene layers or by using a large coupling distance, e.g, when *d* = 50 nm, the “multipole” mode appearances will change into pure dipole mode resonance, as shown in the inserts of Fig. 2e. Besides, we also note that the mode couplings between the two graphene layers are different. Specifically, for the case with *θ* = 0°, the LGNRs-PIT is the result of the strong coupling between LGNRs-0 and UGGs-0, which are resonant along the *x* direction. While for the case with *θ* = 90°, the UGGs-PIT is the result of the strong coupling between UGGs-90 and LGNRs-90, which are resonant along the *y* direction, as clearly shown in Fig. 2. Thus, the LGNRs-PIT and UGGs-PIT are the effects of plasmonic couplings between different modes resonating at different polarizations, resulting in two different PIT effects.

Therefore, we can conclude from Fig. 2 that the LGNRs-PIT (with *θ* = 0°) and UGGs-PIT (with *θ* = 90°) are two different PIT effects resulted from two separate bright to dark mode coupling pathways of the layered graphene system and chartered by the different spectral response. That means we can switch these two PIT effects from one to the other just by changing the polarization direction of the incident light, which is much different from the polarization-insensitive PIT effect (see discussion part). Besides, this switchable PIT effect can be explained by the two-particle model shown in Eqs. (1) and (2). For the case with *θ* = 0°, the direct coupling efficiency of UGGs to the incident field is zero (dark) whereas that of the LGNRs is the highest (bright), as demonstrated by the right-hand side of the equations. While for the case with *θ* = 90°, vice versa, the UGGs become bright whereas the LGNRs are dark. Note that in both cases, the agreement between the analytical results obtained with Eq. (5) (represented by red circles in Fig. 2a, b) and the numerical results (plotted with red lines in Fig. 2a, b) is nearly perfect. Our analytical model predicts very accurately not only the positions but also the peak values of the resonances, as it is clear in Fig. 2. Finally, we note here that the results presented here are much different from other PIT systems that are constructed with the same resonators [35, 38]; this is because they cannot get the results shown in Fig. 2 under different polarizations. We will discuss the differences further in the discussion part.

### Geometrical Tunability of PIT

**Fig. 3:**
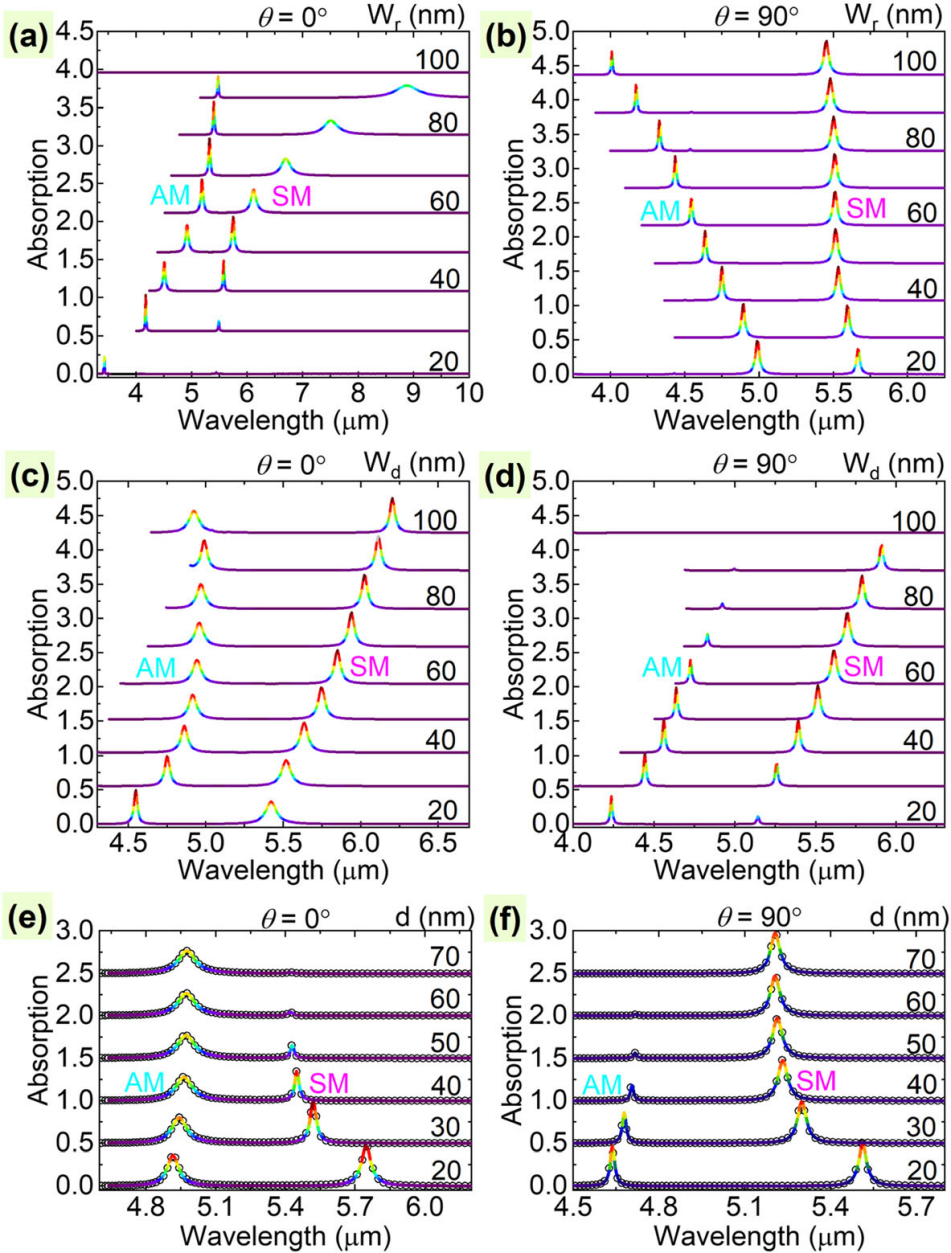
Absorption spectra of the PIT system in the wavelength scale as functions of (**a**), (**b**) the ribbon width *W*_*r*_ of LGNRs; (**c**, **d**) the DG width *W*_*d*_ of UGGs; and (**e**, **f**) the separation distance *d* between the two graphene layers with a step of 10/20 nm at polarization angles of *θ* = 0° (**a**, **c**, **e**) and 90° (**b**, **d**, **f**), respectively. In (**a**–**f**), the deeper red color in the solid lines denotes a stronger absorption. Note that some of the lines are cut to avoid distractions from other higher-ordered absorption peaks. In (**e**) and (**f**), solid curves and dark circles present the numerical and theoretical results, respectively. SM and AM refer to the symmetric mode and antisymmetric mode, respectively

We have demonstrated that the near-field couplings between the bright and the dark modes result in two polarization-dependent PIT effects; therefore, parameters that greatly affect the bright and the dark mode resonances, as well as the coupling strength between them, can be treated as an adjustable parameter for the PIT effects. We first carry out parametric studies for the case with *θ* = 0° by changing the width (*W*_*r*_) of the LGNRs and the widths (*W*_*d*_) of the UGGs from 20 to 100 nm, and show the results in Fig. 3a and c, respectively. Because the LGNRs directly couple with the incident light and work as the bright mode at these conditions, any change in their dimensions directly affects the entire plasmonic response of the system. For example, when *W*_*r*_ is very small, e.g., 20 nm, the coupling efficiency with incident light is very weak due to the low occupation ratio of the GNRs [14, 51], resulting in low absorption of antisymmetric mode and especially the symmetric mode of the PIT system, as can be seen from the absorption line of *W*_*r*_ = 20 nm in Fig. 3a. As another example, when *W*_*r*_ is big enough, and especially when it reaches its maximum 100 nm (that is when the LGNRs is a whole graphene layer), none of the two resonators can couple with the external field and, thus, the LNGRs-PIT disappears. Notably, the absorptions of the two modes show the highest values simultaneously when *W*_*r*_ is around 50 nm. Different from the bright mode, the variations of DG width (*W*_*d*_) in the upper graphene layer for the dark mode can only tune the resonant positions and the absorptions of the symmetric mode and antisymmetric mode within limits, while cannot eliminate or even significantly affecting the high coupling efficiency with the external waves, as shown in Fig. 3c. Actually, even when the dielectric gratings are removed or become a whole dielectric layer (*W*_*d*_ = 100 nm), the LGNRs can still couple with upper graphene sheet, as it has been demonstrated by a two-dimensional case described in a previous work [36], where only one PIT effect is allowed to exist.

However, for the case with *θ* = 90°, the results are in contrast to the case with *θ* = 0° because the LGNRs work as a dark mode while the UGGs work as a bright mode. In detail, the change of the LGNR width *W*_*r*_ only modulates the resonant positions and the maxima absorptions of the symmetric mode and antisymmetric mode, while it cannot extinguish the existence of the two modes, as shown in Fig. 3b. This is because the LGNRs operate as a dark mode in this polarization condition. In the condition with *W*_*r*_ = 100 nm, the system becomes a DG-loaded graphene sheet couples with another graphene sheet, which is similar to a two-dimensional single PIT system reported earlier in another study [34]. However, the change of the upper dielectric gratings will greatly impact the optical response of the PIT system as the upper graphene sheet work as a bright mode in this condition, which is very similar to the case when changing *W*_*r*_ with *θ* = 0°, as shown in Fig. 3d. In general, we can conclude from Fig. 3a–d that the tuning of the bright mode will greatly affect the appearance and even the existence of the UGGs-PIT, as they are demonstrated in Fig. 3a and d, whereas the change of the dark mode can only change the resonant positions and relative strengths of the symmetric mode and antisymmetric mode in the UGGs-PIT, as shown in Fig. 3b and c.

Another parameter that greatly affects the PIT effects is the space *d* between the two graphene resonators. As we fix the widths of the GNRs and dielectric gratings and then increase *d*, the interaction strength between the two graphene resonators decreases monotonically for both polarization angles due to the fast decreasing plasmonic field in the normal direction of the graphene surface [35, 61]. As a result, the symmetric mode and the antisymmetric mode are respectively extinguished for the case with *θ* = 0° and *θ* = 90° at large coupling distance, e.g., *d* > 70 nm, as shown in Fig. 3e and f. As it is known that when the bright and dark modes are far beyond the decay length of the evanescent field of each other, these two modes are uncoupled, and therefore, only the bright mode exists. At that point, we can conclude from Fig. 3e and f that the symmetric mode and antisymmetric mode of the PIT respectively originate from the UGGs and LGNRs, as they remain at large layer distance. Note that the PIT effects at different coupling strengths match well with the two-particle model, as the simulated and analytically predicted results are in excellent agreement, as can be seen in Fig. 3a and b, where the solid curves are gotten from FDTD, and the dark circles are from the two-particle model.

### Electrical Tunability of PIT

**Fig. 4:**
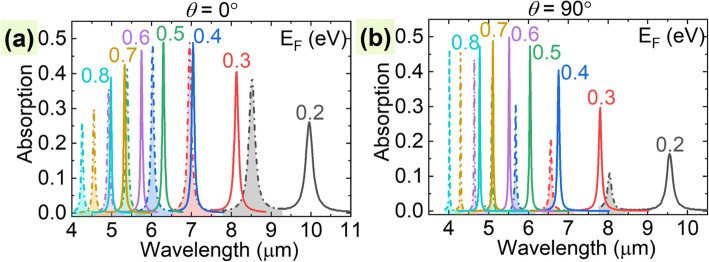
Absorption spectra of the symmetric mode (solid lines) and antisymmetric mode (dash-dotted lines) of the proposed PIT system with different Fermi energy levels of graphene at polarization angles of *θ* = 0° (**a**) and 90° (**b**), respectively

One of the major advantages of graphene-based plasmonic devices is their dynamic and broadband tunability, which can be realized by electrostatic gating techniques [61, 62]. This intriguing property allows us to electrically change the Fermi energy of graphene and, thus, to actively modulate the transmission window of the proposed PIT systems to work at different wavelengths without reconstructing the geometrical structure [24, 25]. By applying different bias voltages with a field-effect transistor structure, researchers have experimentally achieved the dynamical tune of the Fermi energy level from 0.2 to 1.2 eV [63]. The simulated absorption spectra shown in Fig. 4 confirms the broadband and dynamic tunability of the proposed PIT device. For the given geometrical parameters, the plasmon wavelengths of the symmetric mode and antisymmetric mode of the LGNRs-PIT (UGGs-PIT) can be tuned from 4.977 to 9.953 μm and 4.259 to 8.520 μm (from 4.775 to 9.551 μm and 4.015 to 8.033 μm) when the Fermi level is modulated from 0.8 to 0.2 eV, respectively, as the solid and dash-dotted lines shown in Fig. 4a and b. This dynamic tunability will greatly facilitate the design and practical application of the proposed PIT device.

## Applications

In the previous parts, we have made clear how the LGNRs couple with the UGGs and further result in the polarization-dependent PIT effects, and demonstrated how the geometrical and electrical parameters affect the couplings. In this part, we will demonstrate our proposal can be used as selective refractive index sensors and dual-band perfect absorbers.

Considering that the PIT effect is determined by both the bright and dark mode resonances, what brings the change to these two modes will directly alter the symmetric mode and antisymmetric mode in the PIT window. Therefore, the induced symmetric mode and antisymmetric mode are highly sensitive to the local dielectric environment, which can be applied to design refractive index sensors [64]. In our design, both the regions above the UGGs (with refractive index *n*_0_) and below the LGNRs (with refractive index *n*_2_) can be thought of as the sensing regions. To calculate the sensitivities, we define *S* = Δ*λ/*Δ*n*, which specifies the plasmon wavelength (*λ*) shift per refractive index unit (RIU). We assume the refractive indexes of the materials as *n*_1_ = 2.0 and *n*_0_ = *n*_2_ = *n*_3_ = 1.3 (except the cases when *n*_0_ or *n*_2_ is working as the sensing regions with the range changing from 1.0 to 1.1).

**Fig. 5:**
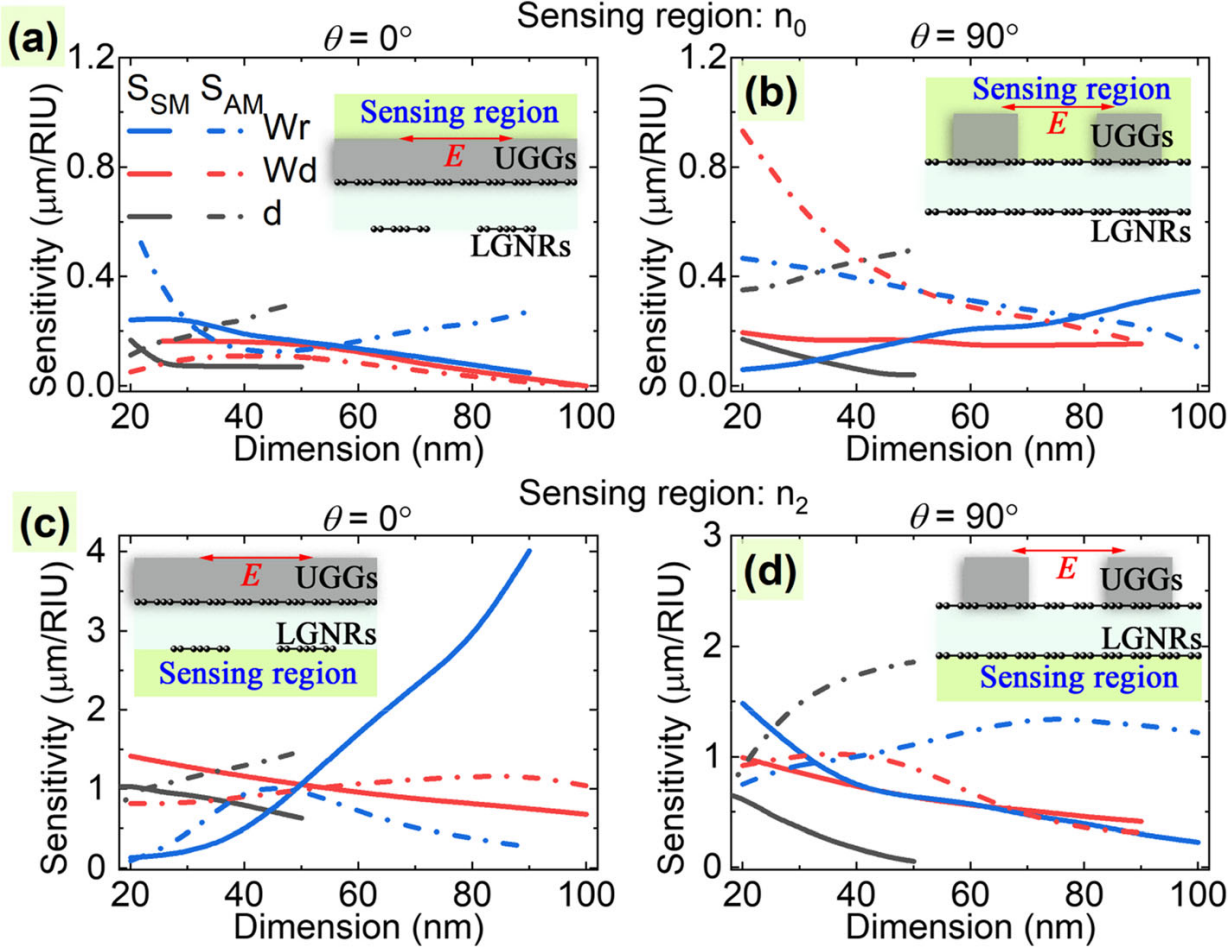
Refractive index sensitivities of the symmetric mode (S_SM_, solid lines) and antisymmetric mode (S_AM_, dash-dotted lines) in the sensing regions of *n*_0_ (**a**, **b**) and *n*_2_ (**c**, **d**) as functions of the ribbon width *W*_*r*_ of LGNRs, the DG width *W*_*d*_ of UGGs, and the separation distance *d* between the two graphene layers at polarization angles of *θ* = 0° (**a**, **c**) and 90° (**b**, **d**), respectively. The inserts show the location of the sensing region

Firstly, when tuning the width of the GNRs (*W*_*r*_), we find that when the sensing region is alongside the bright mode (that are sensing region *n*_0_ with *θ* = 90° and sensing region *n*_2_ with *θ* = 0°), the sensitivity of the symmetric mode *S*_SM_ gets bigger at wider ribbon width (see the solid blue lines in Fig. 5b, c). Especially, *S*_SM_ can reach 4 μm/RIU for the case with *θ* = 0° in sensing region *n*_2_. Secondly, for the case with increasing DG width (*W*_*d*_), both the sensitivity of the symmetric mode *S*_SM_ and antisymmetric mode *S*_AM_ decrease in most cases. Finally, as for the coupling distance *d* between the two layers, it is found that *S*_SM_ decreases while that of the antisymmetric mode increases for both of the polarization angles (see the solid and dash-dotted dark lines in Fig. 5). Considering that the antisymmetric mode will disappear under large coupling distance at the polarization angle of *θ* = 90° (see Fig. 3f), the antisymmetric mode for the situation with *θ* = 0° is more suitable to work as a sensor at a larger distance. Generally, the sensitivities of the symmetric mode and antisymmetric mode of the LGNRs-PIT and UGGs-PIT are respectively comparable to each other, as can be concluded by comparing Fig. 5a with 5b, and Fig. 5c with 5d, respectively. Besides, it is also found that the sensitivities for the cases with sensing regions alongside the bright and dark modes do not show a big difference, as can be seen by comparing Fig. 5a with 5d (alongside the dark mode), and Fig. 5b with 5c (alongside the bright mode). However, the sensitivities of the case with the sensing region under the LGNRs are obviously higher than that of the case with the sensing region above the UGGs, as they are shown by comparing Fig. 5a and b with Fig. 5c and d. This is because the sensitivity is directly related to the localized plasmonic field [64], and the local plasmonic field in the cutting-edge nanoribbons is generally stronger than the continuous edge-free graphene dielectric gratings [60].

**Fig. 6:**
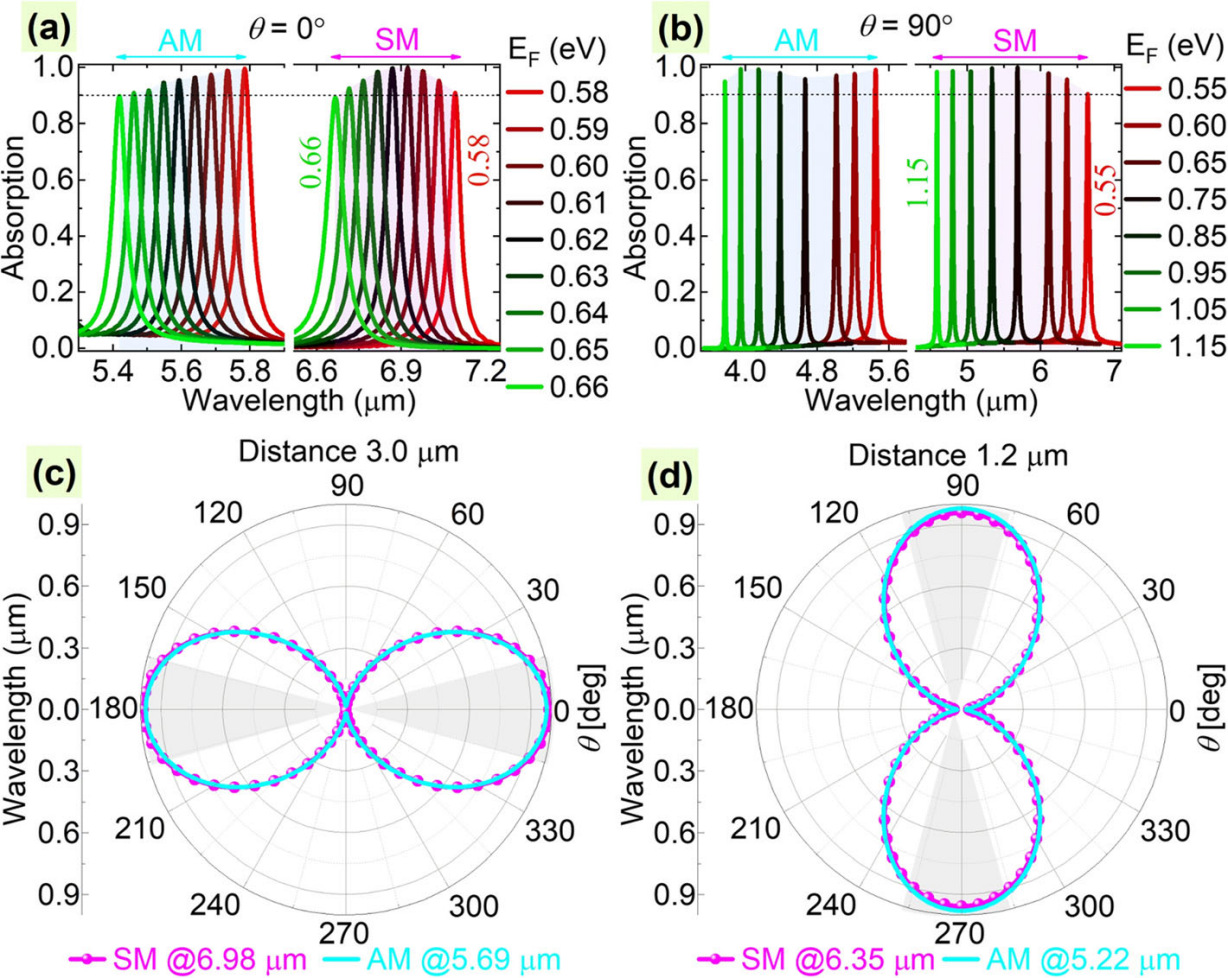
Absorption spectra with different Fermi energy levels of graphene at polarization angles of *θ* = 0° (**a**) and 90° (**b**) for the cases with a metal substrate below the LGNRs with a distance of 3.0 μm (**a**, **c**) and 1.2 μm (**b**, **d**), respectively. (**c**, **d**) Absorption maxima as functions of *θ*. SM and AM refer to the symmetric mode and antisymmetric mode, respectively

Besides working as a refractive index sensor, the proposed system can also be further designed as a perfect absorber. To achieve this, we can add a metallic substrate below the LGNRs and assume the refractive indexes of the materials as *n*_1_ = 2.0 and *n*_0_ = *n*_2_ = *n*_3_ = 1.3. With the existence of the metallic substrate, the dielectric layer between the LGNRs and metallic mirror forms a Fabry-Perot cavity, which can increase the interaction of incidence with graphene layers and further increase the absorptivity of the two modes. For the LGNRs-PIT case with *θ* = 0°, we find that perfect absorptions with absorptivity > 96% of the symmetric mode and antisymmetric mode can be achieved simultaneously when the metallic substrate is with a 3.0-μm distance below the LGNRs, as shown in Fig. 6a and c. We also find that our proposal has good robustness to the doping level of graphene, as shown in Fig. 6a. The absorptivity of the two modes is > 90% when the Fermi energy level of graphene ranges from 0.58 to 0.66 eV. Besides the doping level of graphene, the perfect absorptions also show good tolerance to the polarization angle: The absorptivity of the two modes can keep at a high level (>90%) even the polarization angle ranges from − 17 to 17°. The robustness to the parameters is good for the practical design of the absorber.

To achieve the perfect absorption for the UGGs-PIT case with *θ* = 90°, we need to set the metallic substrate with a 1.2-μm distance below the LGNRs. It is found that perfect absorptions with absorptivity > 95% of the symmetric mode and antisymmetric mode can be achieved simultaneously, as shown in Fig. 6b and d. Similar to the LGNRs-PIT case, it also found that the perfect absorptions show good tolerance to the polarization angle ranging from − 15 to 15° with absorptivity of the two modes > 90% (see Fig. 6d). More notably, the proposed absorber for the UGGs-PIT case shows much bigger robustness to the doping level of graphene, as plotted in Fig. 6b. It is found that the absorptivity of the two modes is > 90% even the Fermi energy level of graphene ranges from 0.55 to 1.15 eV. Considering that the Fermi level of graphene can be dynamically tuned by an external gate voltage, the designed structure can be thought of as active dual-band perfect absorber with a working wavelength of the symmetric mode (antisymmetric mode) ranging from 4.59 to 6.64 μm (3.77 to 5.45 μm).

## Discussions

**Fig. 7:**
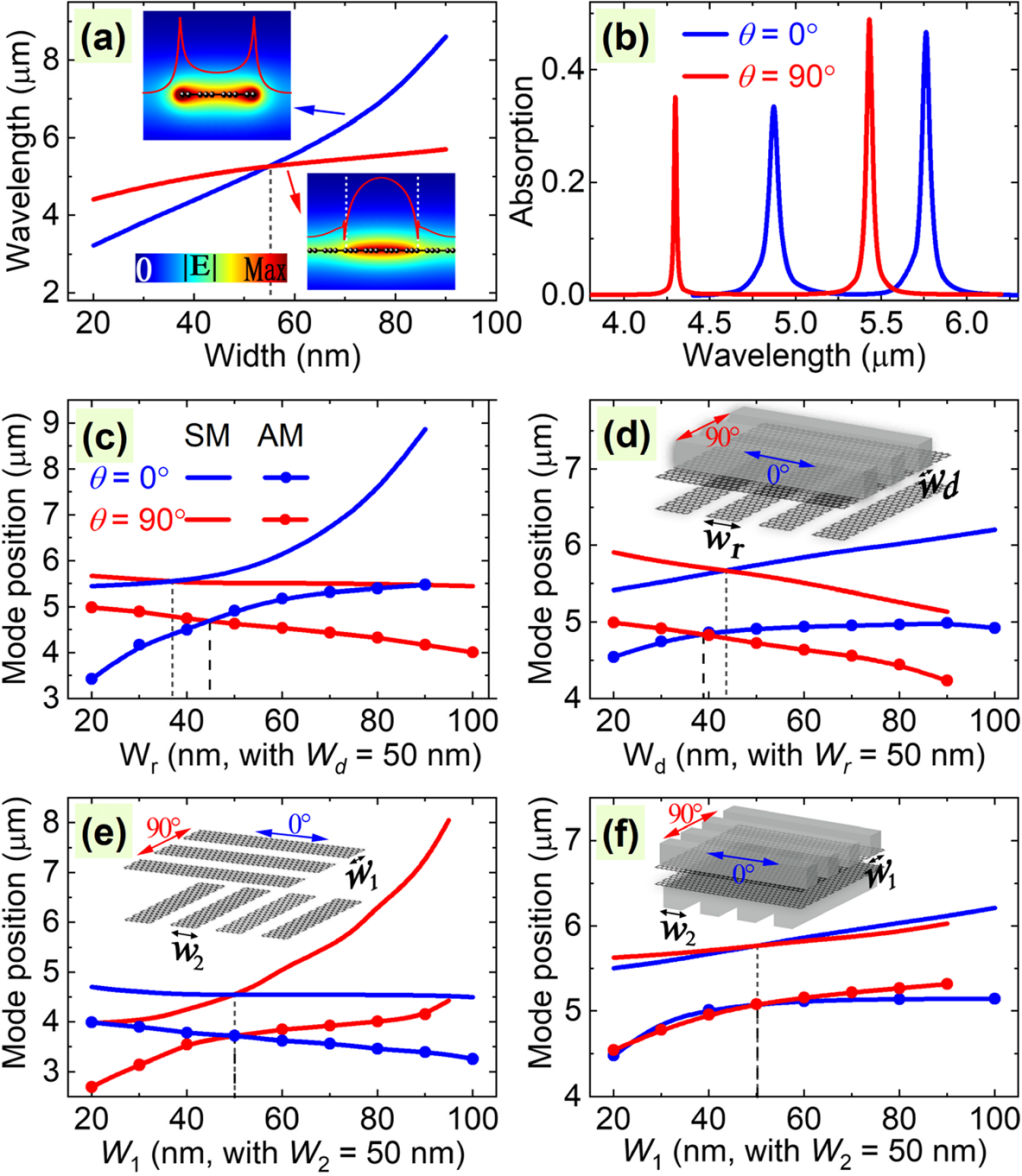
(**a**) Plasmon wavelengths of the cases with only the GNRs and only dielectric grating-loaded graphene sheet for different ribbon and grating width. The inserts show the field distribution of the modes. (**b**) Absorption spectra for different polarization angles of GNR and dielectric grating-loaded graphene-coupled system with their width of 54 nm. (**c**–**f**) Resonant positions of the two PIT peaks at different polarization angles for different systems. The inserts show the corresponding structures

In this part, we discuss the advantages and differences of the proposed structure with other similar structures. To this end, we first calculated the plasmon resonant wavelengths for the cases with only the GNRs and only the dielectric grating-loaded graphene, as shown in Fig. 7a. It shows that the plasmon wavelengths have different dependencies on the width of the resonator. Besides, the inserts show the resonant property of the modes: For GNRs, the plasmonic fields are mainly localized on the edge of the GNR, while for the case with graphene sheet attached with dielectric gratings, the plasmonic fields are mainly concentrated on the grating area. Previous studies have shown that the field distributions and the distance between the resonators will greatly affect the plasmonic couplings [35, 65] and, therefore, the spectral response of the coupled system. That is to say, in our cases, the couplings from the LGNRs to the upper dielectric gratings are different from the other way coupled from the upper dielectric gratings to the LGNRs. Therefore, we obtain the results shown in Fig. 7b that even when the plasmon wavelengths of the two resonators are the same when they exist alone, they will also lead to two distinct PIT effects no matter what they work as bright or dark modes. To show more clearly the advantage of our design, we plot the resonant mode positions of the PIT effects for different geometrical parameters in Fig. 7c and d. They clearly demonstrate that there are always two distinguishable PIT effects for the two polarization directions, even when the geometrical parameters are the same.

However, one may want to know why the proposed structure is not designed with the same resonators, such as perpendicular GNRs and dielectric grating-loaded graphene, as it has been reported in the previous studies [35, 38]. To explain this, we have plotted the resonant positions of the two modes in the PIT effects for the structures with pure GNRs and dielectric grating-loaded graphene resonators in Fig. 7e and f, respectively. It is found that when the two layers of resonators are designed with the same geometrical parameters, there is only one PIT effect for all the polarization directions, which means the PIT effect becomes indistinguishable from the absorption spectrum. This is because the couplings between the two layers of resonators are equivalent due to the same field distribution of the plasmon modes. That is to say, the polarization-independent PIT effects of the structures shown in the insert of Fig. 7e and f depend on the particular choice of the geometrical parameters. Whereas, on the contrary, the design in this paper to achieve two switchable PIT effects is not dependent on the particular choice of the geometrical parameters, which can guarantee the existence of the two switchable PIT effects.

## Conclusion

In this paper, both advanced simulations and theoretical analyses are combined to investigate switchable PIT effects in two graphene layers formed by GNRs coupled with a dielectric grating-loaded graphene layer. Thanks to the crossed nanoribbon and grating directions, both the GNRs and the dielectric gratings can operate as either the bright or the dark mode depending on the polarization direction. The incident light under these two polarization directions introduces two different bright to dark mode coupling pathways within the two resonators, resulting in two switchable PIT effects. Geometrical parameters, such as graphene nanoribbon width, dielectric grating width, layer distance, and graphene Fermi level, are used to study the physical mechanism and the performance of the proposed PIT effect. Additionally, the proposed concepts are examined by applying a two-particle model, showing outstanding agreement with the numerical results. The proposed methods provide a general approach to achieving switchable PIT effects in distinct resonator-coupled system and can advance the applicability and versatility of PIT-based plasmonic sensing platforms and active dual-band perfect absorbers.

## Data Availability

All data supporting the conclusions of this article are included within the article.

## Abbreviations

FDTD: Finite-difference time-domain
GNRs: Graphene nanoribbons
LGNRs: Lower graphene nanoribbons
PIT: Plasmonically induced transparency
UGGs: Upper graphene gratings
