# Correction to: Two Switchable Plasmonically Induced Transparency Effects in a System with Distinct Graphene Resonators

**DOI:** 10.1186/s11671-020-03385-y

**Published:** 2020-08-05

**Authors:** Jingrui Guan, Shengxuan Xia, Zeyan Zhang, Jing Wu, Haiyu Meng, Jing Yue, Xiang Zhai, Lingling Wang, Shuangchun Wen

**Affiliations:** Key Laboratory for Micro/Nano Optoelectronic Devices of Ministry of Education & Hunan Provincial Key Laboratory of Low-Dimensional Structural Physics and Devices, School of Physics and Electronics, Hunan University, Changsha, 410082 China

**Correction to: Nanoscale Res Lett 15, 142 (2020)**

**https://doi.org/10.1186/s11671-020-03374-1**

Following publication of the original article [1], the authors reported an error in Fig. 6; in the Y-axis of Fig. 6c and 6d, it says ‘Wavelength (μm)’ instead of ‘Absorption’.

Please be referred to the corrected figure in this article.

**Fig. 6:**
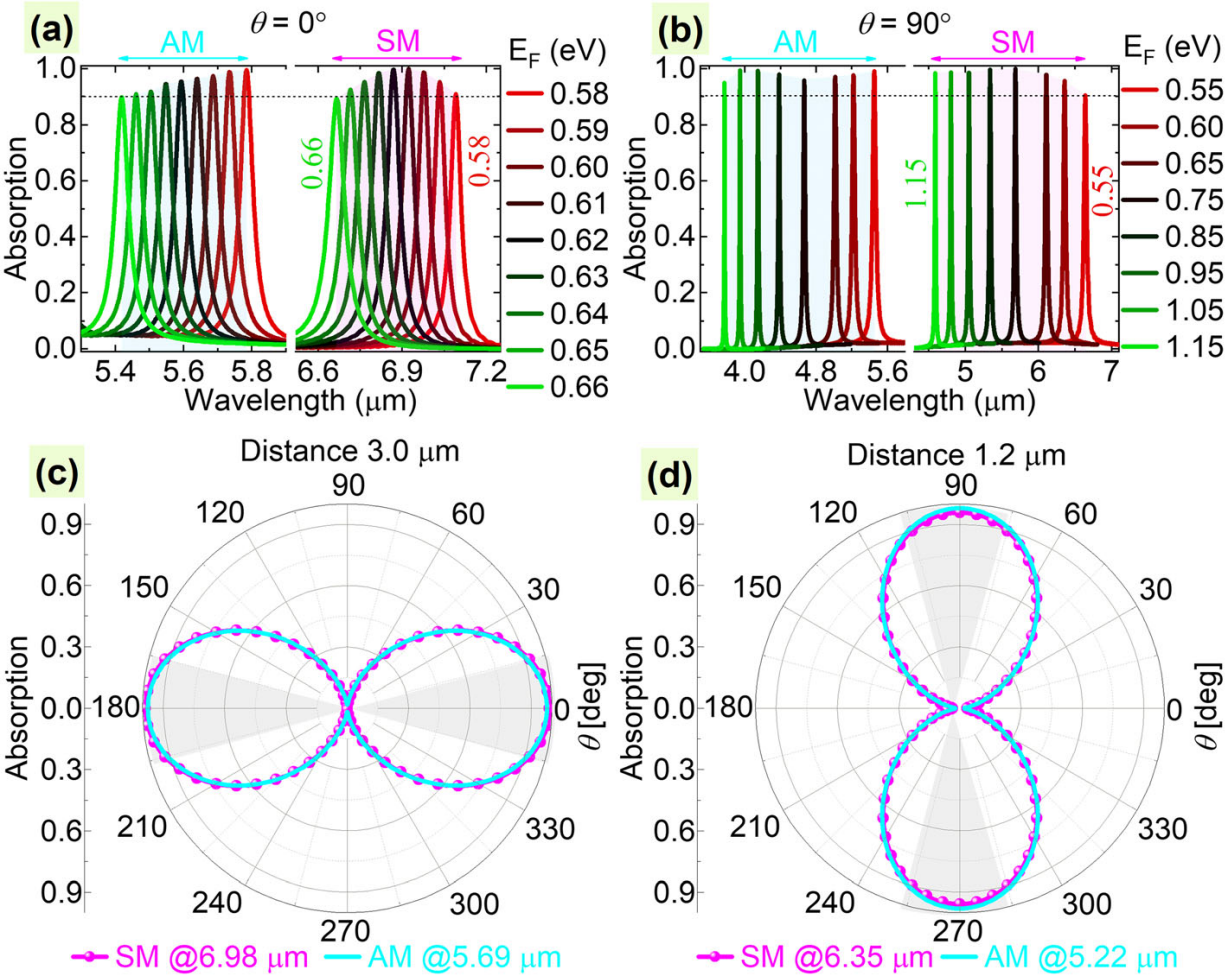
Absorption spectra with different Fermi energy levels of graphene at polarization angles of *θ* = 0° (**a**) and 90° (**b**) for the cases with a metal substrate below the LGNRs with a distance of 3.0 μm (**a**, **c**) and 1.2 μm (**b**, **d**), respectively. (**c**, **d**) Absorption maxima as functions of *θ*. SM and AM refer to the symmetric mode and antisymmetric mode, respectively

The authors apologize for any inconvenience caused.
